# Aroma, Quality, and Consumer Mindsets for Shelf-Stable Rice Thermally Processed by Reciprocal Agitation

**DOI:** 10.3390/foods9111559

**Published:** 2020-10-28

**Authors:** William R. Dixon, Blanca E. Morales-Contreras, Manoch Kongchum, Zhimin Xu, Dustin Harrell, Howard R. Moskowitz, Louise Wicker

**Affiliations:** 1 School of Nutrition and Food Sciences, Louisiana State University Agricultural Center (LSU AgCenter), Louisiana State University, Baton Rouge, LA 70803, USA; William.dixon@purina.nestle.com (W.R.D.); zxu@agcenter.lsu.edu (Z.X.); 2 Nestlé Purina PetCare Company, 1 Checkerboard Square 2N, St. Louis, MO 63164, USA; 3 National Technology of Mexico/I. T. Durango, Graduate in Biochemical Engineering Felipe Pescador 1803, Nueva Vizcaya, Durango 34080, Mexico; bmorales@itdurango.edu.mx; 4 H. Rouse Caffey Rice Research Station, Louisiana State University Agricultural Center (LSU AgCenter), Baton Rouge, LA 70578, USA; mkongchum@agcenter.lsu.edu (M.K.); dharrell@agcenter.lsu.edu (D.H.); 5 Mind Genomics Associates, Inc., White Plains, NY 10605, USA; mjihrm@gmail.com

**Keywords:** amylose, 2-acetyl-1-pyrroline, reciprocal agitation, thermal processing, conjoint analysis, delta E

## Abstract

Food engineering, food chemistry, and consumer segmentation were used to evaluate ready-to-eat rice. The aromatic Louisiana Clearfield Jazzman (CJ) and Thai Jasmine (TJ), and a non-aromatic parboiled (PB) rice were hydrated during the first 10 min of processing with reciprocal agitation followed by static retort processing. The aroma compound, 2-Acetyl-1-pyrroline (2-AP) was more heat-stable in CJ than TJ rice but decreased 15-fold compared to the rice cooker method. Pareto analysis indicated that rice type and agitation had the main effect on amylose and total starch and chroma and hue. Color differences of rice agitated during hydration and between rice cooker or static retort processed rice, indicated only slight differences for each rice variety. Hydration of dry rice during retort cooking and similar starch, color, and aroma quality were achieved with reciprocal compared to static or rice cooker methods. Survey responses categorized consumers into three, mindsets driven by rice consumption, convenience, or packaging.

## 1. Introduction

Rice (*Oryza sativa* L.) is a staple carbohydrate source for billions of people. The impact of rice on food security is clear from production and consumption data. Global production of milled rice exceeded 503.9 million tonnes in 2018/2019 [1] and per capita consumption was 53.9 kg [1]. To improve food security and reduce food waste, strategies were proposed to increase: transparency in food systems, innovations in food manufacturing and packaging, shelf life, and meet consumer needs [2]. The advantages of inclusion of staple foods in a sustainable, consumer-friendly presentation are suggested from life cycle assessment and greenhouse gas emission estimation across food systems [3] and strategic action plans that promote a food systems transformation towards sustainable nutrition [4].

Cultural preferences governing what makes ‘good’ eating quality of rice vary worldwide [5,6]. Several factors affect the eating quality and nutritional benefits of freshly cooked rice, such as rice variety, milling, pre-wash/soaking, and cooking. The amount and type of starch, gelatinization temperature, starch leaching, and swelling, are among the factors that influence rice texture [7]. Stickiness and hardness are fundamental characteristics to describe rice texture, and while amylose is a key determinant of rice-eating quality, other molecular composition and ultra-structure of starch also contribute to palatability, digestibility, and sensory attributes [8,9]. The molecular structure of non-amylose starch also influences rice texture [10] and in rice varieties with similar amylose content, greater amounts of lower molecular mass and smaller shape amylopectin negatively influences quality [8].

Inclusion of staple foods, improvements in health attributes of staple foods, and removal of barriers to consumption of healthier foods contributes to improved nutrition and food security. For example, consumers who included rice in meals also consumed more vegetables, dietary fiber, and iron and consume less energy from fat and saturated fat in their diets [11]. Rice varieties differ in glycemic response, but processing improves the health benefits of rice. Resistant starch increases in retort processed rice with the addition of organic and amino acids [12] or with the addition of xylanase and parboiling pre-treatments [13]. Reduced protein Immunoglobulin G (IgG) response [14] and improved sensory attributes of brown rice [15] are reported in retort rice compared to rice cooker rice.

The time to prepare freshly cooked rice varies from 15 min to 45 min depending on the variety and cooking method, which may be a limiting factor in rice consumption. The growth in convenience rice was attributed to time-challenged urban consumers [8] and a preference shift to better eating quality rice, aroma, texture, ease of preparation, and long retention after cooking [16]. New main dishes and cuisine pairings that feature diverse cuisines offer the opportunity to catalyze the growth of the stagnant rice market if healthier attributes are leveraged. In the U.S. and worldwide, cooking behavior dramatically shifted during the Covid-19 pandemic and consumers made meals from scratch (49%), used precooked and packaged foods (46%), were willing to spend 15–30 min (46%) or 30–60 min (44%) to cook, and ate meals immediately (84%) [17]. The overwhelming avoidance of leftovers, the use of pre-prepared foods, and low time commitment suggests the need for quick, easy to prepare, portion-controlled meals.

The use of convenience rice, such as instant or parboiled, reduces cooking time, but eating quality is lower [5,15]. Limitations of ready-to-heat-and-eat, retort processed rice include off-flavors, off-colors, loss of desirable aroma and flavor, high adhesiveness, and poor eating quality [15]. Rice varieties containing medium to high amylose that exhibit intermediate gelatinization temperatures are better suited for retort operations [8] but palatability may suffer [9]. Sensory attributes of retort rice processed at higher temperatures and shorter times were more similar to home-cooked rice [18].

The quality and nutrition of ready-to-eat retort rice are improved by the use of multiple processes and package technologies. Thermal processing induced deterioration of rice quality was reduced by microwave-assisted thermal processing [19] and high hydrostatic pressure-assisted processing [20]. Packaging materials for ready-to-eat rice include cans [8,21,22] or pouches [18,23]. Most retort studies used static retort processing, but one study used a water spray rotary retort [13].

Rotary retorts and retorts with other forms of agitation improve thermal process parameters, reduce energy use, and improve food quality; reciprocal agitation is more effective compared to static or rotary retorts [24]. Improved process parameters at reciprocal agitation between 60 and 180 shakes per min (SPM) or about 2–3 Hz, or lower speeds of gentle motion of less than 60 SPM, about 1 Hz, were effective for a variety of liquids, purees, or particulates in liquids [25]. For all retort types, changes in retort design, automated computer control, and developments in software allow refinement of individual retort step processes, reduce energy and water use, and reduce over-processing.

Preparation of rice for retort processing included no pre-cooking, partial pre-cooking or fully cooking rice by either water absorption or in excess water, which reduces quality due to full or partial gelatinization prior to retort processing [16]. Reciprocal agitation has the potential to optimize the uniformity of rice hydration and prevent undesirable rice cook qualities [26]. Total thermal load and hold periods may be reduced by packing dry rice and water directly into a container. However, adequate hydration is essential to prevent cracked and undercooked rice kernels; adequate suspension of rice kernels prior to gelatinization is needed to avoid non-uniform cooking.

Innovations in retort design and control, heat penetration data collection, and retort compatible packaging have the potential to create higher quality ready-to-eat meals. Ready-to-eat rice presents a food engineering challenge due to hydration and gelatinization properties. A second challenge to the successful adoption of ready-to-eat rice is the lack of knowledge on consumer mindsets about ready-to-eat rice. Conjoint analysis is a statistical tool to identify aspects of a topic that drives consumer interest and how consumers vary in response to the topic [27]. The conjoint analysis identified consumer mindsets and messaging for insect-based protein [27], effective food safety educational programs [28], messaging to increase fruit and vegetable consumption [29], and messaging to identify acceptable healthier fried chicken alternatives [30]. The objectives of this study were to determine the potential benefit of reciprocal agitation on heat penetration and rice quality, using semi-rigid plastic retort trays with three rice varieties—Clearfield Jazzman CLJ01 (CJ), Thai Jasmine (TJ), and parboiled (PB) rice—to identify consumer segments and drivers around cooking methods, rice use, packaging types, and convenience. The results have an application to improvement in the technology to improve the quality of ready-to-eat rice and to develop strategies to identify key variables relevant to consumers’ preferences for healthier food.

## 2. Materials and Methods

### 2.1. Source of Material

Clearfield Jazzman, CLJ01 (Reg. No. CV-152, PI 687361), (CJ), Thai Jasmine (TJ), (Product of Thailand, Walong Marketing Inc., Buena Park, CA, USA), and Uncle Ben’s parboiled (PB) (MARS, Rancho Domingues, CA, USA) rice were evaluated. Louisiana State University (LSU) H. Rouse Caffey Rice Research Station (Rayne, LA, USA) donated the CJ rice, and PB and TJ rice were purchased from a local grocery store. An aliquot of 128 g (±1 g) dry rice was added to a rigid Silgan package (inner dimensions: 12.5 × 9.5 × 3 cm; Woodland Hills, CA, USA). An aliquot of 141 g (±1 g) of deionized water was added and packages were sealed with a semi-automatic Control GMC PL200G (Boucherville, QC, Canada), using Bemis retort grade L7288 film (Neenah, WI, USA). The settings included a heat seal of 204 °C, a film length of 15.2 cm, a nitrogen gas flush duration of 1.5 s, and a gas pressure of 241 kPa. The same rice/water ratio was used to cook rice in an AROMA electric rice cooker (San Diego, CA, USA).

### 2.2. Retort Preparation and Setting

Rice was processed with a multimode Allpax R&D Retort 2402 Series with Shaka^®^ technology (Covington, LA, USA) in water spray-mode under static (0 SPM) and reciprocal agitation speeds of 45, 90, and 130 SPM. Packaged rice/water was agitated during come up and held 10 min at 60 °C, below the gelatinization temperature, based on literature citations for gelatinization temperature and preliminary estimates of hydration. For each retort run, 10 samples were prepared, which included four thermocouple-probed samples and six non-probed samples. Each retort run was duplicated. TechniCAL CALSoft 5 software and CALPLex data logger (Metairie, LA, USA) were used to track heat penetration curves from probed containers with live tracking of F_0_ values during retort thermal processing at a reference temperature of 121.1 °C and a z-value of 10 °C. Thermocouples were purchased from Ecklund-Harrison Technologies Inc. (Fort Myers, FL, USA).

Data from the slowest heat penetration run at each retort process, or 0, 45, 90, or 130 SPM, was used to determine Ball Formula Method variables (j_h_, f_h_, Bb and Pt), where j_h_ is a dimensionless number that represents the heating lag rate factor during come-up and f_h_ is the time to increase the temperature by 1 log during the cook. Ball’s cook time (Bb) and Ball’s cook time with come-up time credit [Pt = Bb − (0.42 × CUT)] were determined at F_0_ of 6. All F values were based on a reference temperature of 121.1 °C and z = 10 °C. The initial temperature (IT), retort temperature, and come-up-time (CUT) were set to 20 °C, 121.1 °C, and 13.5 min, respectively, to determine Bb and Pt cook times. The critical factors included a maximum product fill weight of 271 g, an orientation of the longest package length placed in direction of reciprocation of retort containers, and thermocouple position at the geometric center of containers.

### 2.3. Rice Starch and Proximate Analysis

Prior to analysis, a center cut of the rice packages was made with a stainless steel rectangular cutter (11.3 × 7.4 × 5 cm). The center cut rice was frozen at −20 °C and freeze-dried (Genesis Pilot Lyophilizer, Ridge, NY, USA) for 5 days. The freeze-dried rice was ground with a stainless steel Waring blender and sieved through a 250-micron mesh screen, and stored at −20 °C until analysis.

The starch analyses were duplicated from replicate retort runs or electric rice cooker batches. Amylose, resistant starch, non-resistant starch, and total starch were determined for CJ, PB, and TJ rice using Megazyme assay kits, K-AMYL-AM/AP, K-RSTAR, and KTSTA-100A, respectively, (Chicago, IL, USA).

Proximate analysis was measured for electric rice cooker and retort processed, 130 SPM, CJ rice (LSU Agricultural Chemistry, Baton Rouge, LA, USA) and included protein, crude fat, ash, carbohydrates, moisture and mineral analysis (e.g., phosphorous (P), iron, (Fe), boron (B), calcium (Ca), copper (Cu), magnesium (Mg), manganese (Mn), potassium (K), sodium (Na), sulfur (S), and zinc (Zn)) of rice. Moisture (Ohaus MB45 Moisture Analyzer) was measured in duplicate for retorted rice CJ, TJ, and PB, electric rice cooker rice, and retort processed at 0, 45, 90, and 130.

### 2.4. Rice 2-AP Aroma Analysis

The aroma compound, 2-AP was measured by an automated headspace gas chromatography technique [31] in duplicate, only for the aromatic rice, CJ and TJ, using freeze-dried samples as described earlier [32,33]. An aliquot of 1000 g of ground samples was placed in 20 mL headspace glass vials. An aliquot of 1 μL of 0.5 mg/mL of 2,6-dimethylpyridine (2,6-DMP) was added to each vial before airtight sealing with a polytetrafluoroethylene/silicone septum secured by an aluminum seal cap. 2-AP was measured with a Shimadzu GC-2010 Plus system connected with headspace autosampler model HS-20 (Shimadzu, Columbia, MO, USA) coupled to a flame thermionic detector. The concentration of 2-AP was identified by the gas chromatography retention times relative to the 2-AP standard that was run under the same conditions. Peak areas were obtained with the aid of LabSolutions software (Shimadzu, Columbia, MO, USA).

### 2.5. Rice Color

L*a*b* values were determined using the CIE lab-scale (Konica Minolta CR-5 Chroma Meter, Ramsey, NJ, USA). Polystyrene disposable plastic Petri dishes (Ø = 35, H = 10 mm) were filled with five-gram aliquot of center-cut rice and evenly distributed with a spatula to cover the entire dish diameter and ensure the same height. The color was measured six times for each retort and electric rice cooker process based on the average of 2 scans for each color measurement.

The chroma (C, Equation (1)), hue (h*, Equation (2)), ΔE referencing difference from electric rice cooker (ΔE_RC_, Equation (3)), and ΔE referencing difference from 0 SPM (ΔE_0SPM_, Equation (4)) were determined from L*a*b* values [34].

(1) $$C=\sqrt{a^{*=}+b^{*=}}$$

(2) $$hue^{*}=arctan2\left(b^{*}/a^{*}\right)$$

(3) $${\Delta E}_{\mathrm{RC}}=\sqrt{{\left(L^{*}-L_{\mathrm{RC},\mathrm{Avg}}^{*}\right)}^{2}+{\left(a^{*}-a_{\mathrm{RC},\mathrm{Avg}}^{*}\right)}^{2}+{\left(b^{*}-b_{\mathrm{RC},\mathrm{Avg}}^{*}\right)}^{2}}$$

(4) $${\Delta E}_{\mathrm{SPM}}=\sqrt{{\left(L^{*}-L_{0\mathrm{SPM},\mathrm{Avg}}^{*}\right)}^{2}+{\left(a^{*}-a_{0\mathrm{SPM},\mathrm{Avg}}^{*}\right)}^{2}+{\left(b^{*}-b_{0\mathrm{SPM},\mathrm{Avg}}^{*}\right)}^{2}}$$

### 2.6. Package Oxygen and Carbon Dioxide Levels

Residual oxygen and carbon dioxide in the headspace of retort containers were measured using a five-sec draw through a rubber septum (Systech Illinois GS6600 O_2_ & CO_2_, IL, USA). A total of two containers were analyzed for each speed (0, 45, 90, and 130 SPM). Nitrogen gas flush was set to achieve a target oxygen headspace less than 1%.

### 2.7. Consumer Survey

The Mind Genomics approach, a conjoint analysis procedure, was modified from procedures described by Saulo and Moskowitz and Dinasco et al. [28,30]. In the current study, four questions were generated, each with four messages (elements) for a total of 16 elements, and each element represented a specific piece of information. The elements constituted responses to each of four questions about cooking methods, the inclusion of rice in meals, the convenience of ready-to-eat rice, and package, respectively. Each respondent evaluated a set of 24 unique vignettes, viz., combinations of 2–4 elements, combined by the experimental design. The ratings were converted to a binary scale (positive vs. not positive feeling). The data was used to create an individual-level model for each respondent, with a clustering program dividing the respondents into non-overlapping groups, based upon the pattern of coefficients for the model relating the presence/absence of the elements to the ratings. The ratings were transformed to a binary scale (1–6 transformed to 0; 7–9 transformed to 100). Clustering by the respondents by the pattern of their coefficients generated mindsets. High scoring elements were defined as those elements showing a coefficient of +12 or higher from regression, either for total panel or for a mindset.

The experiment was implemented using commercially available software (www.BimiLeap.com). Respondents were recruited using Lu.cid Marketplace Services under a BimiLeap User agreement. Participants were selected from southeastern states in the U.S.A. (Alabama, Arkansas, Florida, Georgia, Kentucky, Mississippi, North Carolina, South Carolina, and Tennessee) with an income between $20,000 and $69,999 and were not age-restricted.

Panelists gave informed consent before participation in the study. The protocol was approved by the LSU Agricultural Center Institutional Review Board (HE20-27). Panelists were asked ‘How likely are you to buy and use ready-to-heat-and-eat rice?’. Participants also answered an additional question about cooking during Covid-19, with four possible responses. Categories from each element were presented on the screen in a series of vignettes and participants evaluated the entire vignette on a 9-point scale, with 9 representing very likely and 1 representing not at all likely.

### 2.8. Statistical Analysis of Rice Physico-Chemical Properties

Statistical analysis of heat penetration data was performed using JMP Pro 14 (Cary, NC, USA) statistical software for Ball factors using linear regression analysis to determine significance at 5%; mean and standard deviation were determined for all treatments from triplicate replications. A full factorial design was used to analyze the effect of rice type (CJ, PB, and TJ) and agitation (0, 45, 90, 130 SPM) during the cooking process on rice quality characteristics of starch and color parameters. The results were analyzed by Dunnet’s multiple comparison test with RC-controls, with significance at 5%, to analyze different treatments with respect to the RC sample for each type of rice. The Minitab Software version 18 (Minitab Inc., PA, USA) was used.

## 3. Results

### 3.1. Rice Heat Penetration and Ball Factors

**Figure 1:**
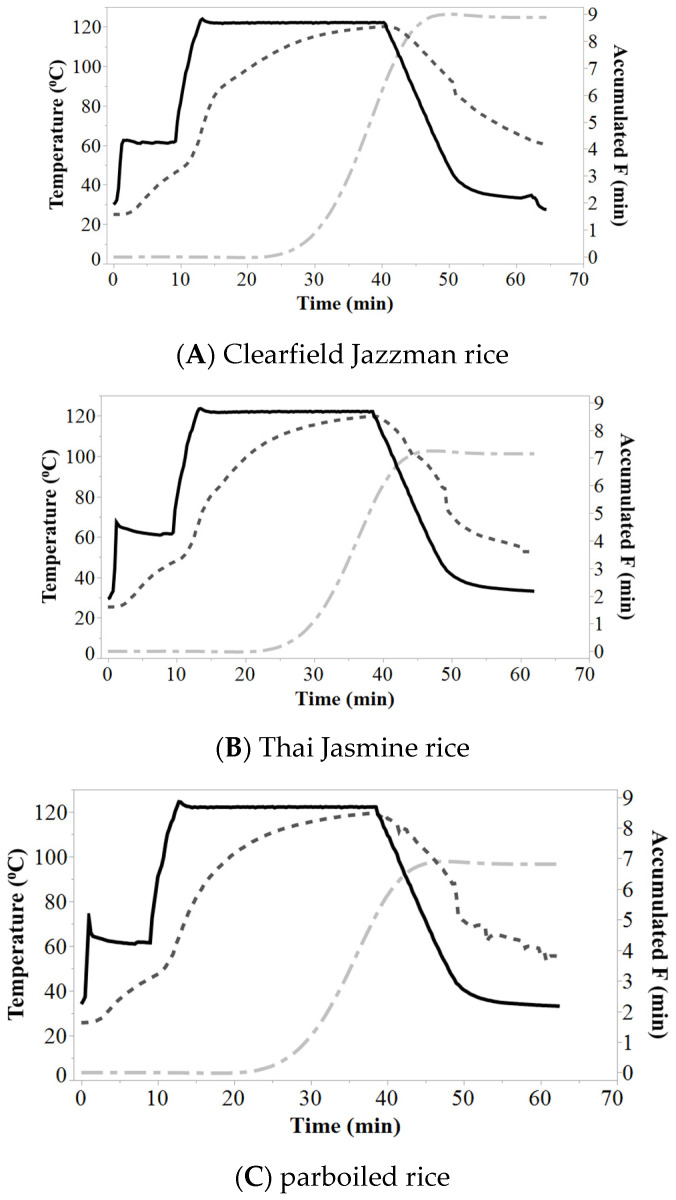
Clearfield Jazzman (**A**), Thai Jasmine (**B**), and parboiled (**C**) rice slowest heat penetration data at 0 SPM displaying retort temperature (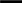), heat penetration (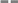), and accumulated F_0_ (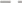). shakes per min (SPM).

**Figure 2:**
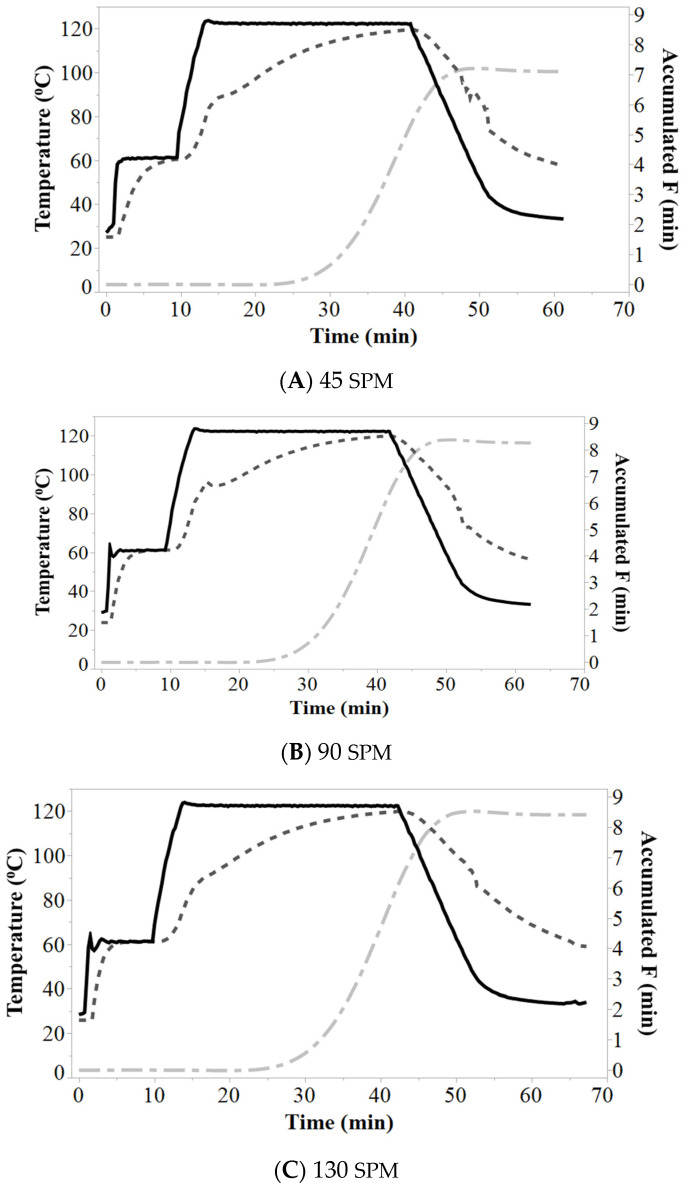
Clearfield Jazzman (CJ) rice slowest heat penetration data at 45 (**A**), 90 (**B**), and 130 (**C**) SPM displaying retort temperature (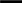), heat penetration (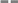), and accumulated F_0_ (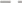).

Heat penetration was determined for all retort runs and data was presented for the slowest probe of heat penetration with no reciprocal agitation of CJ, TJ, and PB rice (Figure 1A–C). The heat penetration of the product temperature lags the retort temperature during come-up, cook and cool for all rice, and the heat penetration profiles appear similar to slower heat penetration after the apparent gelatinization of rice starch during the cook and cool. The heat penetration profile with reciprocal agitation of 45, 90, and 130 SPM for CJ rice showed that with increasing reciprocal agitation, the temperature increased more rapidly with agitation during the 10 min hold for CJ rice (Figure 2A–C). A similar trend was observed for TJ and PB rice (Supplementary Figures S1 and S2). The retort temperature was held at 60 °C, a temperature lower than gelatinization, with reciprocal agitation to enhance hydration prior to gelatinization and potentially shorten the processing time. The product and retort heat penetration data were more closely overlayed with increased reciprocal agitation during the first 10 min of processing (Figure 2A–C) compared to static (Figure 1A). However, 90 and 130 SPM had minimal differences in heat penetration during come-up. Reciprocal agitation provided quicker heat transfer to the product during come-up and hold. After reciprocal agitation halted, heat penetration was similar. Subsequent gelatinization of rice resulted in heat transfer by conduction. Heat transfer was not improved if reciprocal agitation was continued during the cook and cool. The F_0_ values from the slowest heat penetration data ranged between 7 and 9 min for CJ rice, above the target of 6 min, attributed to the challenges to estimate the time to enter and exit cool with rice gelatinization.

The slowest heat penetration data and the corresponding Ball factors for each rice type and each reciprocal agitation speed are depicted in Table 1. Data were normalized to F_0_ of 6 for comparison. The Heat F ranged from 5.00 to 6.40 min, the Cool F ranged from 1.45 to 2.73 min, and Total F ranged from 6.41 to 8.87 min. Variations in target F_0_ might be related to variations in the rice, especially parboiled rice, degree of hydration, starch gelatinization, and heat penetration after gelatinization.

The f_h_ values for the slowest heat penetration data for CJ, TJ, and PB are presented in Table 1. The f_h_ values ranged from 18.6 and 22.3 min and were similar to f_h_ values of 17.5 to 26 min reported for Jasmin, Reungkeaw, and Pinkeaw rice [22]. The j_h_ values for the current study ranged between 0.94 and 1.25 (Table 1). Similar j_h_ values of 0.44 and 1.17 were reported for a rice-kheer, pudding type product, packaged in a pouch, and processed in a rotary retort [35]. For CJ and PB rice, the j_h_ values decreased (*p* < 0.05) and the f_h_ values increased (*p* < 0.05) with agitation, indicating the beneficial effect of agitation during the 10 min hold.

The cook times (Bb) for the slowest heat penetration data ranged from 32.1 min for TJ-0, to 35.3 min for CJ-130), but there were no significant differences (*p* > 0.05) in Bb between 0 and 130 SPM for the rice. If come-up-time is taken into consideration, all cook times could be reduced by over 5 min (Table 1).

### 3.2. Rice Starch Characterization

The amylose, resistant starch, non-resistant starch, and total starch for each retort processed rice are reported in Table 2. Rice cooked in an electric rice cooker served as control. The amylose content of CJ-90 or CJ-130 rice was 26.6 g/100 g and 34.7 g/100 g and significantly higher (*p* < 0.05) than amylose in CJ-RC at 18.8 g/100 g. See bolded values that highlight significant differences. The amylose content of CJ-0 and CJ-45 was not significantly different from CJ-RC. Amylose content in the current study was higher than the value of 12.8 g/100 g amylose for CJ [36]. Amylose content of TJ or PB retort processed rice was not different from the control rice cooker rice (Table 2). The amylose content of uncooked CJ, TJ, and PB rice was 14, 17, and 36 g/100 g, respectively. Cooking rice resulted in an increase in amylose for all cook processes for all rice. Retorted TJ rice and PB rice, purchased at a retailer, had an amylose content of 34 g/100 g and 36 g/100 g, respectively, and was within the range of this study.

For each rice type, the resistant starch, non-resistant starch, and the sum were not significantly different. The starch analysis suggests a minimal effect on starch composition by retort processing compared to electric rice cooker prepared rice. PB rice had consistently higher values for resistant starch than CJ or TJ rice. In the direct measure of total starch (TS), there was a significant increase (*p* < 0.05) in total starch in PB-130 relative to PB-RC. Overall, the resistant starch values between 1.29 and 1.87 g/100 g, were slightly lower in the current study than reported for retort processed brown rice of 2.03 and 2.54 g/100 g [13]. Increased resistant starch was attributed to the hydrothermal process and pre-gelatinization of starch [37]. The direct measure of total starch of most rice ranged between 60 and 72 g/100 g. The sum of resistant starch and non-resistant starch closely approximated the empirical measure of total starch within 10 g/100 g, with an exception for CJ-0, PB-130, and PB-45 rice with no discernible trend.

**Figure 3:**
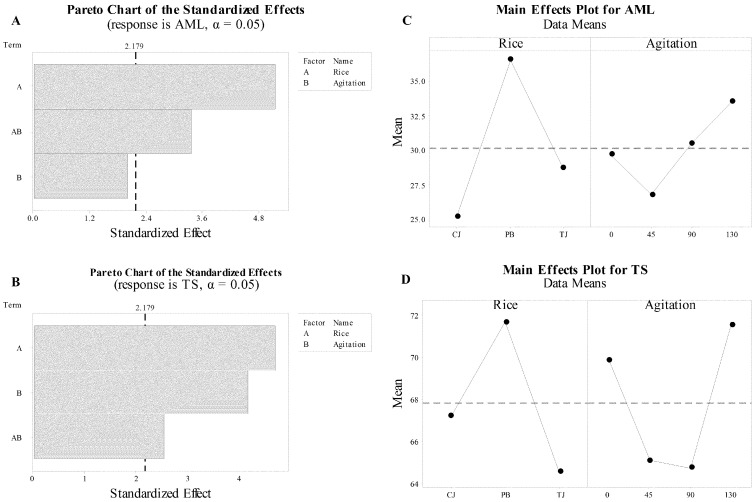
The analysis of the full factorial design presented as Pareto and main effects plots for amylose (AML: (**A**,**C**), respectively) and total starch (TS: (**B**,**D**) respectively).

The main effects on starch derived from the full factorial design analysis and the absolute values of the standardized effects from the largest to the smallest effect are depicted using Pareto charts for amylose and total starch (Figure 3A,B). The reference line (dotted line) indicates which effects were statistically significant. Rice type and interaction of rice type and agitation showed a significant effect on amylose and the contribution of rice type had greater magnitude than interactive effects. The effect of agitation was not significant. For total starch, rice type, agitation, and interaction, all had a significant (*p* < 0.05) effect (Figure 3B).

The main effect plots complement the Pareto charts for amylose and total starch (Figure 3C,D). The dotted reference line represents the average of the response. The trend of rice and agitation is not parallel to the X-axis, which shows the main effect of these parameters on amylose. The main effects plot shows that PB rice had a positive effect on amylose and total starch in comparison with aromatic rice (CJ and TJ, Figure 3C,D), and reciprocal agitation tends to increase amylose; however, the change in the magnitude of the response is not large enough to be significant. In the main effects plot for total starch, there is no consistent trend to describe total starch fluctuations with reciprocal agitation; PB tends to have a higher total starch effect than CJ or TJ (Figure 3D).

### 3.3. Rice Aroma Intensity

The 2-AP content of CJ-RC was 0.776 ppm and is more than twice that of TJ-RC at 0.271 ppm (Table 2). Retort processing reduced the 2-AP values by 10 to 100 fold in CJ or TJ rice, respectively, relative to CJ-RC or TJ-RC (Table 2). Retention of 2-AP in the retort rice of this study was improved relative to commercial ready-to-eat Thai Jasmine rice, which had undetectable levels of 2-AP. Japonica rice, cooked with high-pressure steam up to 0.18 MPa, retained up to 3190 ppm 2-AP and showed potential for improving the eating quality of rice, including other flavor compounds, texture color, and sensory attributes [38]. 2-AP is produced in the rice kernel and a key indicator of rice aroma [39]. Greater 2-AP retention suggests a more buttery popcorn aroma profile [40]. The aroma of rice is a particularly important quality attribute and increasingly popular with consumers and part of the local and national identity [16], but the volatile aroma compound, 2-acetyl-1-pyrroline (2-AP), is heat-sensitive [39].

### 3.4. Rice Color Properties

The color parameters of L*, a*, b*, hue, and chroma, represent lightness to darkness, red to green, yellow to blue, color perception, and saturation of color, respectively. Colorimeter values are presented in Table 3. The L* values for CJ rice ranged from 71.7 to 76.3 and were not significantly different, regardless of the cooking method. The L* values for TJ rice ranged from 76.0 to 78.7 and higher, for TJ-0 and TJ-130, indicating significantly higher lightness, compared to TJ-RC, TJ-45, or TJ-90. The L* values for PB rice were not different (*p* > 0.05) by the cook methods. The L* values for PB rice ranged from 64.7 to 70.6 and were lower than for CJ or TJ rice, indicating PB was darker rice.

Trends for a* values showed no significant differences by cook method for CJ and TJ rice, but PB rice had significantly higher a* values for PB-45 and PB-90 than PB-0 or PB-130 (bolded). Only PB rice had positive a* values. The b* values for CJ rice ranged from 13.2 to 15.5 and were not significantly different. TJ rice had b* values that ranged from 8.3 to 10.7. TJ-RC and TJ-0 had significantly different b* values (bolded) from TJ-45, TJ-90, or TJ-130. The b* values for PB rice ranged from 20.8 to 22.5 and b* values of PB-45 were significantly higher (bolded) than other PB rice.

Collective changes in L*, a*, and b* can be used to estimate h*, the color perceived by the consumer and C, the saturation of color. The h* and C values for CJ rice were not significantly affected by the cooking method. The h* and C values of TJ rice showed that retort cooking and agitation decreased h* and increased C (*p* < 0.05) (Table 3). Likewise, h* and C of PB rice showed a significant decrease in h* and an increase in C at intermediate to high agitation speeds. TJ rice was the only rice with significantly lower saturation C values for rice cooker than any retort cooked rice (Table 3). The higher b* values in retort cooked TJ likely account for lower C values. Higher b* values indicate a shift towards the yellow from the blue quadrant in the color diagram, resulting from the more severe thermal treatment of the retort and differences in perception of color or colorant compounds in retort cooked rice.

**Figure 4:**
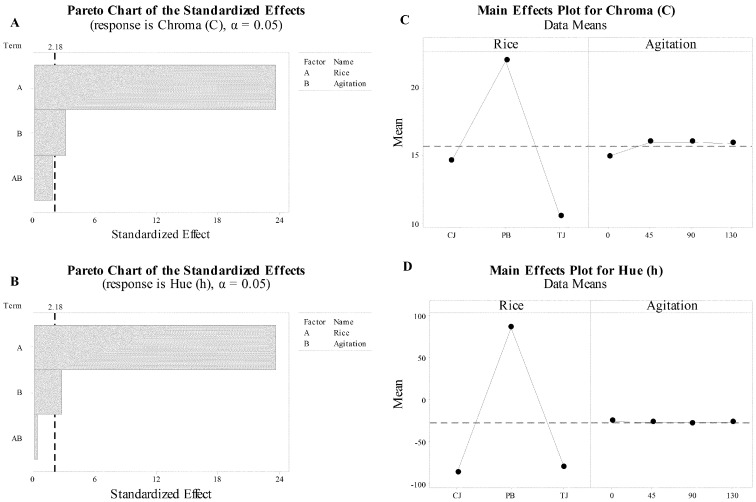
The analysis of the full factorial design presented as Pareto and main effects plots for chroma (C: (**A**,**C**), respectively) and hue (h: (**B**,**D**) respectively).

The main effects of rice type and agitation to chroma are presented in a Pareto chart (Figure 4A). Both have a significant impact, but rice type has a larger significant effect; the interaction effect was not significant. Similar results were observed for the color parameter, h (Figure 4B), and rice type had the largest significant effect. The plot of the main effect showed that the agitation effect was almost parallel with the X-axis, indicating minimal impact and supported that rice type was the main effect for C (Figure 4C) and h* (Figure 4C). The aromatic rice, CJ and TJ, had negative a* and h* values. Since PB rice and aromatic rice cross quadrant boundaries, measuring the relationship between variables is not practical [41]. However, the aromatic rice (CJ and TJ) were close in the pure spectrum of color.

The ΔE_RC_ values are shown for rice cooked in the retort relative to rice cooked in an electric cooker and ΔE_SPM_ values of rice cooked by retort with agitation relative to rice cooked with a static process (Table 3). The ΔE_RC_ values for CJ rice relative to the electric rice cooker ranged between 4.1 and 4.7. The ΔE_RC_ values for TJ or PB rice relative to the electric rice cooker were 2.3 to 3.5 and slightly lower than observed for CJ rice. The ΔE_SPM_ values of retort processed by agitation for CJ, TJ, or PB rice was less than 2.0, excepting CJ-90 at 2.4+, indicating non-perceptible differences in retort processed rice. The greater the ΔE values, the greater the difference in perception of color. The ΔE value of 2.0 or less is considered the threshold for perception of color differences to consumers; tolerance for instrumental color differences may be established by calibration against a standard, but ΔE values between 2 and 10 are considered perceptible at a glance [42].

The L* values for PB rice in this study were approximately ten units higher and b* values were 5 units higher in b* than parboiled rice processed in pouches [13]; color differences are presumably due to the source of the parboil, retort method, and package. L*, a*, and b* of retort processed Thai Jasmine flour were similar to the present study [43]. Unlike the present study, L* values decreased with pressure in Japonica rice. The b* values ranged between 8.3 to 15.5 and were higher than b* of high pressure steamed Japonica rice with b* values of 4.31 and 7.97.

### 3.5. Rice Proximate and Mineral Composition

Proximate and mineral analysis of CJ rice was determined at 0 SPM to describe the nutritional content (Supplementary Table S1). Compared to Jasmin rice [44], CJ rice had more protein, lipids, and carbohydrates. The CJ rice had less fiber and higher amounts of micronutrients, potassium, salt, and iron [44]. Moisture content range between 49 and 59% for all rice samples. Arsenic levels in CJ rice were less than 0.165 mg/100 g, the limit of detection for the instrument. Arsenic levels of 0.02 mg/100 g were reported for basmati, jasmine, parboiled, and white rice [45].

### 3.6. Container Headspace

Carbon dioxide was higher than oxygen in all containers of rice. There were no differences in rice container headspace for oxygen. Oxygen levels were less than 1% in CJ, TJ, and PB rice. In commercial packages of ready-to-eat Thai Jasmine and parboiled rice processed in pouches, the oxygen exceeded 12%, indicating the probable lack of a nitrogen gas flush.

### 3.7. Consumer Response to Ready-to-Heat-and-Eat Rice 

A total of 199 respondents included 70 males and 129 females. Selected respondents were restricted to specific states in the southeastern U.S. and with an annual household income between $20,000 and $69,999. The population in these southeastern U.S. states have higher incidences of obesity and effective intervention trials are needed. The income of the target population for intervention is above the poverty line and above and below-median incomes for the targeted states, representing broad segments of the population. The orientation page, classification, and additional questions are provided in Table 4. Here, the purpose of the study, the process of the survey, and how the data will be used, are described. A summary of the responses to the classification question and consumer mindsets are provided in Table 5. The responses are categorized into three distinct mindsets that are almost evenly distributed at 31 to 34% of respondents. The data shows positive coefficients for the total and for each mindset, with ‘strong performing’ operationally defined as a coefficient of +10 or higher. A high positive coefficient indicates the element is a strong driver to convince respondents to eat the product [27].

Based on the strong performing elements, Mindset 1 could be described as PackagingPhiles. Elements with packaging terms, including steel cans, rigid plastic, and flexible pouches, generated strong interest. None of the elements pertaining to rice preparation, meals with rice or convenience, resonated strongly with this group. The PackagingPhiles are strongly motivated by all messages regarding recycling, re-use, and single-use packaging.

Mindset 2 could be described as the Busy Rice Lovers who hate to cook. This group responds strongly by messaging which talks about the inclusion of rice in meals and that rice represents an easy meal, never boring and satisfying. They are not motivated, but not averse to convenience rice.

Mindset 3 might best be described as Easy Meal Rice Cooks and are more conventional in meal planning. This group is strongly motivated by portion-controlled microwaved rice as well as cooking and leftover rice. Convenience, re-use, recycling are nearly neutral terms with the Easy Meal Rice Cook Mindset.

Prior to the Covid-19 pandemic, many meals were consumed away from home from a variety of food-service outlets, such as restaurants, fast food carry-out, and grocery deli section carry-out. In response to the additional question, ‘How do you feel about cooking and eating at home during Covid?’, 124 of the 199 respondents selected, ‘I love having more time to plan and cook meals at home.’ A lot fewer, 36 of 199, selected, ‘I like to cook, but I don’t always have what I need in my kitchen’, and 24 of 199 selected, ‘I cook at home more now but it is frustrating if no one likes it.’ The selection to ‘I cook at home, but it takes a lot of planning and preparing; I prefer carry-out or restaurants’ generated the lowest number of respondents at 16 of 199. The overwhelmingly favorable response about cooking at home and a very low response to preference for restaurants or carry-out meals strongly contrasts with consumer behavior before the Covid-19 pandemic. The pattern of responses suggests that cooking at home is preferred to restaurant meals when the barriers to cooking and meal preparation are removed.

## 4. Discussion and Conclusions

In this study, feasibility was demonstrated for the preparation of quality, ready-to-eat aromatic rice without previous cooking or hydration using reciprocal agitation retort processing. Dry rice was hydrated and retort processed within the sealed package. Reciprocal agitation decreased the temperature differential between retort and product temperatures during the hydration step at 60 °C and temperature differential varied with reciprocal agitation speed and rice type. Reciprocal agitation facilitated in-package hydration and maintained rice quality. Further optimization of hydration conditions, specific to rice cultivars, would likely improve the uniformity of hydration, gelatinization, and eating quality of retort rice. An additional limitation of the study is the absence of an evaluation of the textural quality of the retorted rice. Instrumental and sensory assessment of texture is needed for the full application of the technology. Cultural and regional preference must be identified that influence liking of rice textures from sticky to individual kernel. The aroma of CJ and TJ rice was adversely affected by retort processing. Minimal changes in quality of starch physico-chemical characteristics or color attributes were observed for retort processed rice relative to electric rice cooker rice. The concept of ready to heat and eat rice is not new. Mind Genomics addresses the issue of understanding specific messages, ideas, and communication which interest the respondent. Mind Genomics indicates that consumers distribute into groups embracing traditional methods of cooking rice, easy meals, or driven by packaging and not the rice product. Awareness of drivers and detractors of consumer choice of processed foods, assist in the further development of viable, innovations in thermal processing methods, packaging choices, and ready-to-eat food products. Development of long shelf life, high-quality foods that are part of a healthy diet addresses sustainable nutrition and food security.

## Figures and Tables

**Table 1:** Ball factors and cook times for three rice varieties at four process speeds using slowest heat penetration data ^1,2^.

		Slowest Heat Penetration Data				Ball Formula Factors		Process Time (min) at F_0_ = 6	
Rice	SPM	IT (°C)	Heat F (min)	Cool F (min)	Total F (min)	j_h_	f_h_	Bb	Pt
CJ	0	25.0	6.14	2.73	8.87	1.19	18.6	32.8	27.1
	45	25.0	5.22	1.88	7.10	0.98	21.7	34.9	29.2
	90	23.9	5.93	2.34	8.27	0.94	21.2	34.0	28.3
	130	25.9	5.92	2.49	8.40	0.95	22.3	35.3	29.7
TJ	0	25.4	5.22	1.93	7.15	1.13	18.4	32.1	26.5
	45	25.1	5.00	2.52	7.52	0.98	21.8	35.0	29.4
	90	24.7	6.40	1.96	8.35	1.21	18.9	33.3	27.7
	130	24.7	4.87	1.54	6.41	1.25	18.1	32.6	26.9
PB	0	25.8	5.18	1.64	6.82	1.12	19.4	33.3	27.6
	45	24.8	5.08	2.48	7.56	1.18	19.5	33.9	28.2
	90	25.5	5.81	1.94	7.75	1.08	19.4	33.0	27.3
	130	25.8	5.23	1.45	6.67	0.98	20.9	34.0	28.3

^1^ Abbreviations: Clearfield Jazzman (CJ), Thai Jasmine (TJ), and parboiled (PB), shakes per min(SPM), initial temperature (IT), F_0_ accumulated during come-up and cook (Heat F), F_0_ accumulated during cooling (Cool F), and the sum of Heat F and Cool F (Total F), Ball’s cook time (Bb), Ball’s cook time with come-up-time (Pt). ^2^ Ball formula settings: j_c_ was kept constant at 1.41, f_c_ = f_h_, come-up-time was 13.5 min, IT was 20 °C, and retort temperature was 121.1 °C.

**Table 2:** Starch and aroma quality attributes of rice cooker and retort processed rice ^1,2^.

Sample	AML	RS	NRS	RS+NRS	TS	2-AP
**CJ–RC**	18.8 ^A^ ± 0.00	0.04 ^A^ ± 0.06	68.7 ^A^ ± 6.11	68.8 ^A^ ± 6.17	65.1 ^A^ ± 2.01	0.776 ^A^ ± 0.116
CJ-0	18.3 ^A^ ± 0.01	0.13 ^A^ ± 0.02	64.3 ^A^ ± 1.86	64.5 ^A^ ± 1.84	71.3 ^A^ ± 1.96	**0.083 ± 0.034**
CJ-45	21.2 ^A^ ± 0.01	0.70 ^A^ ± 0.78	69.6 ^A^ ± 9.90	70.3 ^A^ ± 10.68	63.1 ± 4.39	**0.030 ± 0.000**
CJ-90	**26.6 ± 0.01**	0.06 ^A^ ± 0.03	70.0 ^A^ ± 4.67	70.1 ^A^ ± 4.64	63.9 ^A^ ± 1.18	**0.035 ± 0.013**
CJ-130	**34.7 ± 0.06**	0.06 ^A^ ± 0.04	71.1 ^A^ ± 6.37	71.1 ^A^ ± 6.34	70.4 ^A^ ± 2.79	**0.065 ± 0.077**
**TJ–RC**	27.8 ^A^ ± 0.08	0.49 ^A^ ± 0.26	69.5 ^A^ ± 9.55	70.0 ^A^ ± 9.29	69.1 ^A^ ± 4.34	0.271 ^A^ ± 0.012
TJ-0	38.2 ^A^ ± 0.06	0.15 ^A^ ± 0.04	71.6 ^A^ ± 4.34	71.8 ^A^ ± 4.38	69.8 ^A^ ± 3.27	**0.030 ± 0.011**
TJ-45	21.9 ^A^ ± 0.04	0.21 ^A^ ± 0.17	72.6 ^A^ ± 6.13	72.8 ^A^ ± 6.29	61.2 ^A^ ± 3.12	**0.006 ± 0.009**
TJ-90	26.1 ^A^ ± 0.00	0.16 ^A^ ± 0.02	70.7 ^A^ ± 9.20	70.9 ^A^ ± 9.22	62.2 ^A^ ± 3.02	**0.007 ± 0.000**
TJ-130	28.5 ^A^ ± 0.06	0.10 ^A^ ± 0.07	73.0 ^A^ ± 7.67	73.1 ^A^ ± 7.73	65.0 ^A^ ± 2.95	**0.007 ± 0.000**
**PB–RC**	40.1 ^A^ ± 0.09	1.29 ^A^ ± 1.06	63.2 ^A^ ± 3.83	64.4 ^A^ ± 4.89	70.2 ^A^ ± 0.47	
PB-0	32.6 ^A^ ± 0.03	1.52 ^A^ ± 0.40	59.9 ^A^ ± 10.43	61.4 ^A^ ± 10.83	68.4 ^A^ ± 0.62	
PB-45	37.2 ^A^ ± 0.06	1.72 ^A^ ± 0.22	55.3 ^A^ ± 0.45	57.0 ^A^ ± 0.67	71.0 ^A^ ± 3.16	
PB-90	38.8 ^A^ ± 0.02	1.58 ^A^ ± 0.026	60.8 ^A^ ± 16.62	62.4 ^A^ ± 16.36	68.2 ^A^ ± 1.09	
PB-130	37.4 ^A^ ± 0.01	1.86 ^A^ ± 0.30	61.9 ^A^ ± 10.39	63.7 ^A^ ± 10.09	**79.2 ± 0.37**	

^1^ Clearfield Jazzman (CJ); Thai Jasmine (TJ); parboiled (PB) were cooked in a retail rice cooker (RC), in retort without reciprocal agitation (0), in retort at 45 SPM (45), in retort at 90 SPM (90), in retort at 130 SPM (130). AML, RS, NRS, RS+NRS, TS, and 2-AP denote amylose, resistant starch, non-resistant starch, the sum of RS and NRS, total starch, and 2-Acetyl-1-pyrroline, (2-AP), respectively. 2-AP was reported only for the aromatic rice. ^2^ Dunnett’s Multiple Comparisons of retort cooked rice compared to rice cooker (RC)-Controls. Means not labeled with the letter ^A^ on each type of rice group are significantly different (*p* < 0.05) from the respective RC Control and are bolded to enhance clarity.

**Table 3:** Color quality attributes of rice cooker and retort processed rice ^1,2^.

Samples ^1^	L*^2^	a*^2^	b*^2^	h*^2^	C^2^	ΔE_RC_	ΔE_0SPM_
**CJ–RC**	71.7 ^A^ ± 1.13	−1.3 ^A^ ± 0.26	14.2 ^A^ ± 0.53	−84.8 ^A^ ± 1.20	14.2 ^A^ ± 0.50	−	−
CJ−0	75.5 ^A^ ± 0.76	−1.3 ^A^ ± 0.04	13.2 ^A^ ± 0.93	−84.3 ^A^ ± 0.58	13.2 ^A^ ± 0.92	4.1 ± 0.7	−
CJ−45	76.3 ^A^ ± 0.55	−1.0 ^A^ ± 0.06	14.8 ^A^ ± 0.24	−86.2 ^A^ ± 0.17	14.9 ^A^ ± 0.24	4.7 ± 0.5	1.9 ± 0.2
CJ−90	75.7 ^A^ ± 0.23	−0.9 ^A^ ± 0.22	15.5 ^A^ ± 0.22	−86.6 ^A^ ± 0.86	15.5 ^A^ ± 0.20	4.3 ± 0.5	2.4 ± 0.3
CJ−130	74.4 ^A^ ± 3.03	−1.0 ^A^ ± 0.03	14.8 ^A^ ± 0.20	−86.1 ^A^ ± 0.15	14.9 ^A^ ± 0.19	4.1 ± 1.0	1.9 ± 0.3
**TJ−RC**	76.0 ^A^ ± 0.31	−2.3 ^A^ ± 0.05	8.3 ^A^ ± 0.06	−74.5 ^A^ ± 0.19	8.5 ^A^ ± 0.15	−	−
TJ−0	**78.7 ± 0.75**	−2.2 ^A^ ± 0.41	9.7 ^A^ ± 0.32	−77.2 ^A^ ± 2.71	**10.0 ± 0.22**	2.8 ± 0.8	−
TJ−45	77.1 ^A^ ± 0.10	−1.9 ^A^ ± 0.08	**10.3 ± 0.27**	**−79.4 ± 0.70**	**10.5 ± 0.25**	3.2 ± 0.3	1.9 ± 1.3
TJ−90	77.6 ^A^ ± 0.12	−1.8 ^A^ ± 0.10	**10.7 ± 0.27**	**−80.7 ± 0.74**	**10.9 ± 0.25**	2.9 ± 0.7	1.0 ± 0.6
TJ−130	**78.1 ± 0.70**	−2.1 ^A^ ± 0.36	**10.7 ± 0.81**	−79.0 ^A^ ± 1.03	**10.9 ± 0.86**	3.5 ± 0.6	1.1 ± 0.7
**PB−RC**	64.7 ^A^ ± 0.01	0.502 ^A^ ± 0.01	20.8 ^A^ ± 0.19	88.6 ^A^ ± 0.02	20.8 ^A^ ± 0.19	−	−
PB−0	66.9 ^A^ ± 0.32	1.4 ^A^ ± 0.53	21.6 ^A^ ± 0.33	86.3 ^A^ ± 1.34	21.7 ^A^ ± 0.36	3.0 ± 0.8	−
PB−45	65.9 ^A^ ± 1.73	**1.9 ± 0.24**	**22.5 ± 0.71**	**85.2 ± 0.45**	**22.6 ± 0.72**	2.3 ± 0.4	1.8 ± 0.4
PB−90	66.9 ^A^ ± 0.86	**1.8 ± 0.41**	21.5 ^A^ ± 0.53	**85.5 ± 0.64**	21.6 ^A^ ± 0.55	3.0 ± 0.4	1.6 ± 0.3
PB−130	70.6 ^A^ ± 4.58	1.6 ^A^ ± 0.10	21.9 ^A^ ± 0.03	**86.0 ± 0.29**	21.9 ^A^ ± 0.06	3.2 ± 0.9	1.4 ± 0.3

^1^ Clearfield Jazzman (CJ); Thai Jasmine (TJ); parboiled (PB) were cooked in a retail rice cooker (RC), in retort without reciprocal agitation (0), in retort at 45 SPM (45), in retort at 90 SPM (90), in retort at 130 SPM (130). L*a*b* represents CIE color parameters; ΔE_RC_ represents the perceived color difference between the rice cooker and retort processed rice. ΔE_0SPM_ represents the perceived color difference between retort rice processed without agitation to rice cooked with reciprocal agitation. ^2^ Dunnett’s Multiple Comparisons of retort cooked rice compared to rice cooker (RC)-Controls. Means not labeled with the letter A on each type of rice group are significantly different (*p* < 0.05) from the respective RC Control and are bolded to enhance clarity.

**Table 4:** Respondent orientation and classification question.

**A. Respondent Orientation Page.**	
Rice is a staple food. Cooking rice at home takes about 40 min. There are new, pre-cooked rice products that can be stored in your pantry and are ready to microwave and eat in minutes. The pre-cooked rice is in packaging that looks like either a pouch or a bowl. The purpose of this study is to understand some of the factors that go into planning easy, family meals, and how ready-to-eat rice may contribute to healthier meals.	No individual names will be collected. All answers will be summarized only in aggregate form and no individual answers can be identified. You will see a statement and various combinations of phrases. The phrases may seem repetitive, but each combination is unique. Your participation is voluntary and by proceeding through the survey, you consent to participate. You may stop at any point in the survey.
**B. Classification question.**	
We want to develop more nutritious foods that you like to eat, that fit in your lifestyle, that are more sustainable, and to develop highly effective nutrition education programs. How likely are you to buy and use ready to heat and eat rice?	Answer: 9 (likely) to 1 (unlikely).
**C. Additional optional question.**	
How do you feel about cooking and eating at home during Covid?’	I love having more time to plan and cook meals at home I like to cook, but I don’t always have what I need in my kitchen I cook at home more now but it is frustrating if no one likes it. I cook at home, but it takes a lot of planning and preparing; I prefer carry-out or restaurants.

**Table 5:** Categories and elements and segmentation of consumers into three groups ^a,b^.

			Mindsets		
	Mindsets: 1-PackagingPhiles; 2-Busy Rice Lovers; 3-Easy Meal Rice Cooks	Total	1	2	3
	**Base Size**	199	62	68	68
	**Additive Constant**	52	50	52	52
	**Question A: Methods of cooking, preparation and keeping rice**				
A1	Cook methods: You cook rice for several meals and save cold left-overs to eat later	0	5	−12	6
A2	Cook methods: You cook rice for a single meal	2	4	−7	8
A3	Cook methods: You microwave cooked rice, just enough for each person	1	0	−8	12
A4	Cook methods: You cook rice for several meals and freeze leftovers to eat later	2	−2	−6	13
	Q**uestion B: Reason for rice in meals**				
B1	RTB: Always have ready-to-eat rice in your pantry for a fast, easy meal for your hectic days	4	−2	13	−2
B2	RTB: Rice fills you up when you are really hungry	3	−4	14	−2
B3	RTB: Rice can be served with different recipes and never boring	3	−5	15	−3
B4	RTB: Rice can be served to even the pickiest eater	1	−7	9	−2
	**Question C: Convenience of cooked, ready-to-eat rice**				
C1	Convenience: you can heat and eat in the same package, no cooking, no clean-up	1	−6	1	6
C2	Convenience: you can microwave rice, and the rice is never gummy	2	−1	1	7
C3	Convenience: you can microwave rice and dinner is ready in minutes	3	−4	2	9
C4	Convenience: you can microwave rice, add a stew and have a meal	1	−7	1	8
	**Question D: Package**				
D1	Package: steel can, 100% recyclable, use your own bowl to re-heat	0	12	−2	−9
D2	Package: plastic package, heat and eat, wash and use again	0	14	−5	−8
D3	Package: plastic package, heat and eat, partially recyclable	1	14	−5	−7
D4	Package: flexible pouches, heat and eat, dispose in trash	1	14	−5	−6

^a^ Absolute values greater than 8 indicate drivers of consumer choice (green) or barriers to consumer choice (red). ^b^ Participants were selected from southeastern states in the U.S.A. (Alabama, Arkansas, Florida, Georgia, Kentucky, Mississippi, North Carolina, South Carolina, and Tennessee) with an income between $20,000 and $69,999. Age ranges were 18–24, 25–34, 35–44, 45–54, 55–64, 65+; percentages in each age range were 2.5% 28.6%,22.6%, 16.6% 14.6% 15.1%, respectively.

## Supplementary Materials

The following are available online at https://www.mdpi.com/2304-8158/9/11/1559/s1, Figure S1: Thai Jasmine (TJ) rice slowest heat penetration data at 45 (A), 90 (B) and 130 (C) SPM, Figure S2: Parboiled (PB) rice slowest heat penetration data at 45 (A), 90 (B) and 130 (C) SPM. Table S1: Proximate and mineral analysis of Clearfield jazzman rice retort processed at 0 SPM.
