# Supplementary material for: Aroma, Quality, and Consumer Mindsets for Shelf-Stable Rice Thermally Processed by Reciprocal Agitation
Source: Foods. 2020 Oct 28;9(11):1559. doi: 10.3390/foods9111559 (PMC7692449; doi:10.3390/foods9111559)
Supplement: Supplementary file 1 [file foods-09-01559-s001.pdf]

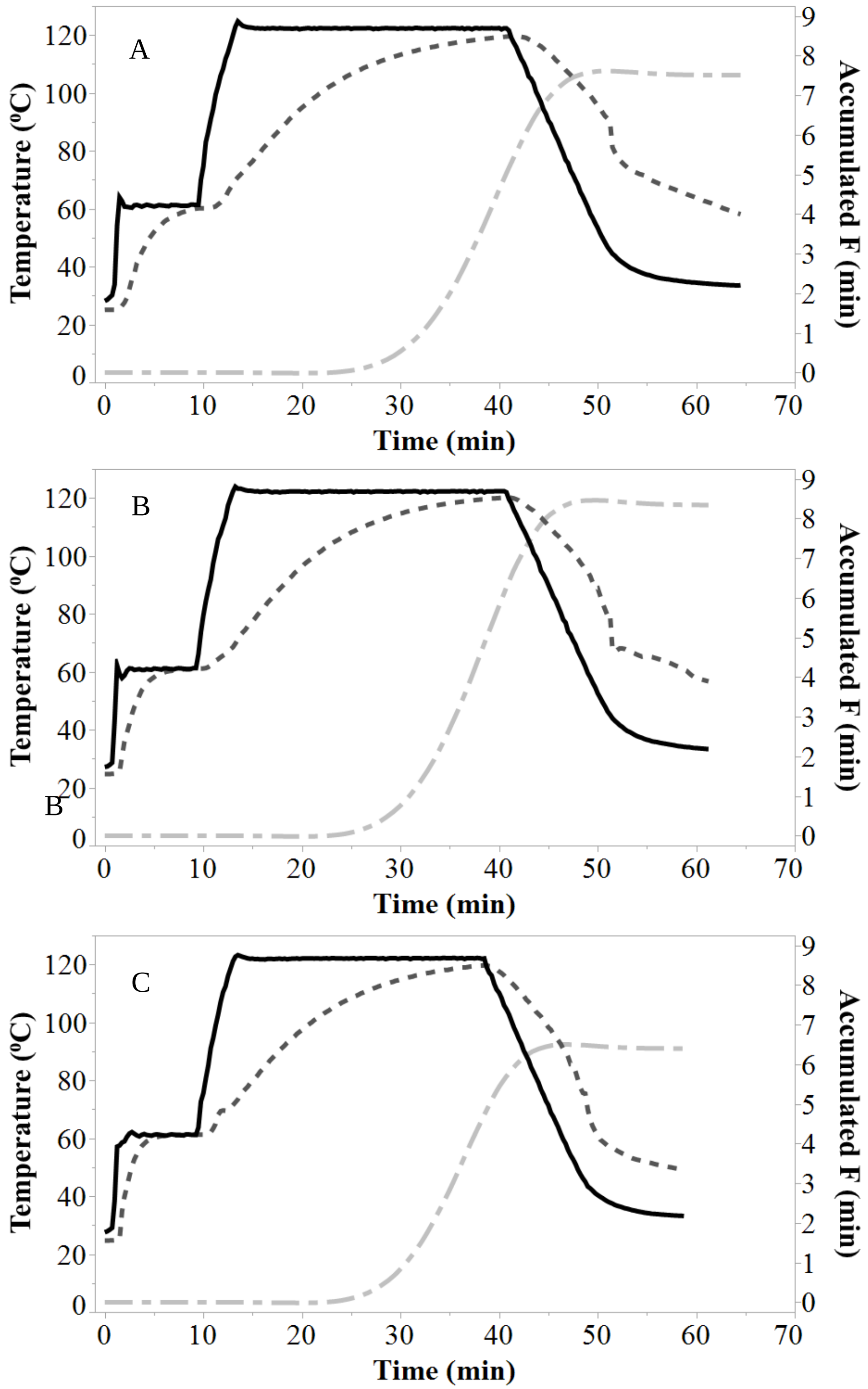

Supplemental Figure 1 A, B, C. Thai Jasmine (TJ) rice slowest heat penetration data at 45, 90 and 130 SPM, respectively, displaying retort temperature (▬), heat penetration (‑ ‑), and accumulated F_0 (‑ ‑).

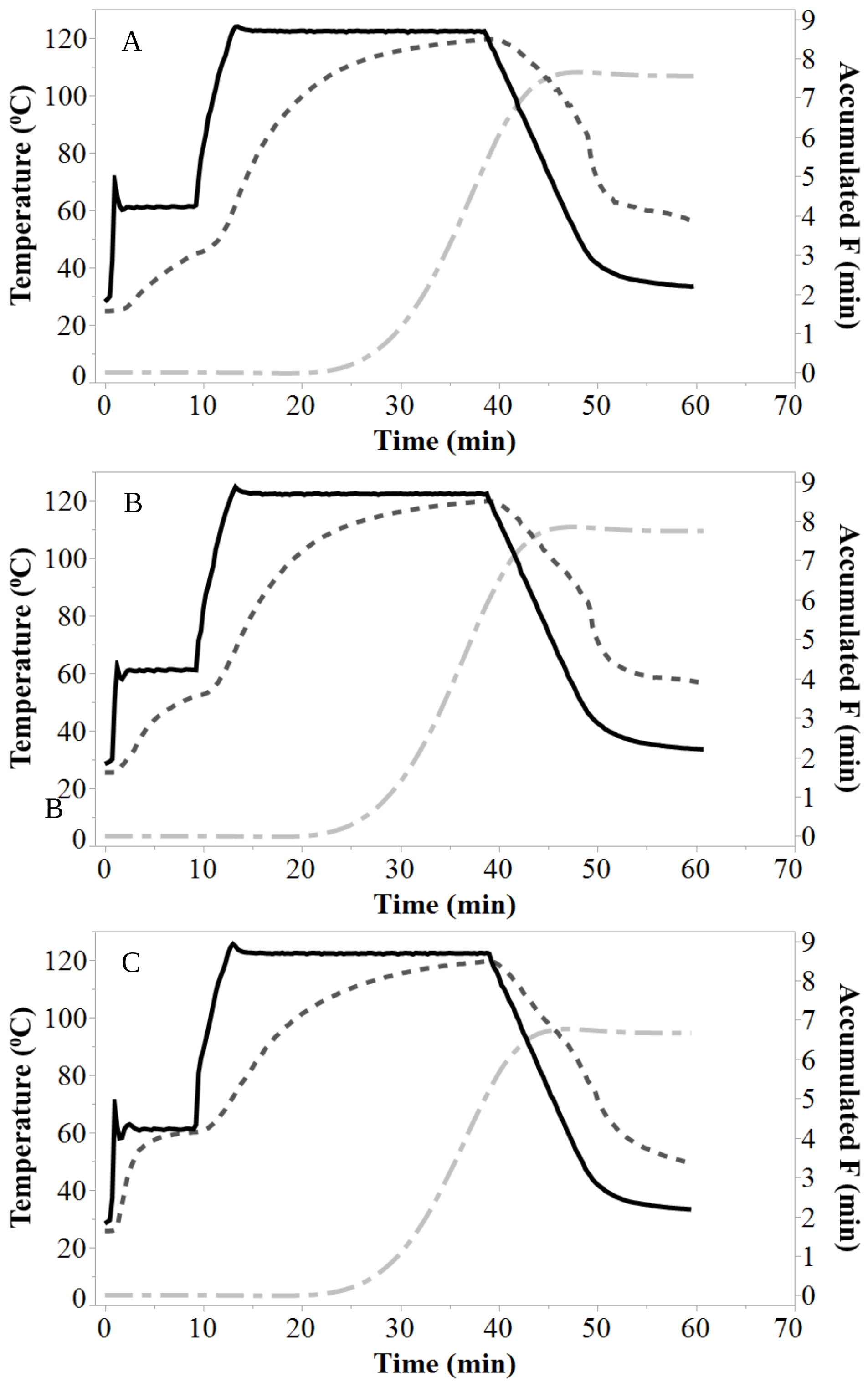

Supplemental Figure 2 A, B, C. Parboiled (PB) rice slowest heat penetration data at 45, 90 and 130 SPM, respectively, displaying retort temperature (▬), heat penetration (- -), and accumulated F_0 (– -).

Supplemental Table 1. Proximate and mineral analysis of Clearfield jazzman rice retort processed at 0 SPM.

Proximate Analysis	
**Proximate**	**Percent (%)**
Protein	3.55% ± 0.07%
Crude Fat	0.17% ± 0.01%
Ash	0.30% ± 0.00%
MC	55.9% ± 1.43%
Carbohydrates	40.1% ± 1.52%
**Mineral Analysis**	
**Mineral**	**CJ Retort Conc (mg/100g)**
Boron	**0.697 ± 0.052**
Calcium	**3.00 ± 0.000**
Copper	**< 0.190 ± 0.028**
Iron	**0.384 ± 0.028**
Magnesium	**0.265 ± 0.035**
Manganese	**0.708 ± 0.020**
Phosphorus	79.50 ± 7.778
Potassium	56.50 ± 6.364
Sodium	< 13.00 ± 1.414
Sulphur	48.50 ± 0.707
Zinc	1.340 ± 0.057
Aluminum	0.805 ± 0.013
Barium	0.057 ± 0.045
Cadmium	< 0.012 ± 0.000
Chromium	0.050 ± 0.018
Cobalt	< 0.008 ± 0.000
Lead	< 0.050 ± 0.000
Molybdenum	0.060 ± 0.008
Nickel	0.029 ± 0.006
Selenium	< 0.575 ± 0.007
Arsenic	< 0.165 ± 0.007
Mercury	0.001 ± 0.000
